# Automated Gravimetric Calibration to Optimize the Accuracy and Precision of TECAN Freedom EVO Liquid Handler

**DOI:** 10.1177/2211068216632349

**Published:** 2016-02-23

**Authors:** Laurent Bessemans, Vanessa Jully, Caroline de Raikem, Mathieu Albanese, Nicolas Moniotte, Pascal Silversmet, Dominique Lemoine

**Affiliations:** 1 GSK Vaccines, Rixensart, Belgium; 2 Neomed Insitute, Laval, Canada.

**Keywords:** High-throughput screening, robotics and instrumentation, instrumentation qualification, systems, visual basic, fixed tips

## Abstract

High-throughput screening technologies are increasingly integrated into the formulation development process of biopharmaceuticals. The performance of liquid handling systems is dependent on the ability to deliver accurate and precise volumes of specific reagents to ensure process quality. We have developed an automated gravimetric calibration procedure to adjust the accuracy and evaluate the precision of the TECAN Freedom EVO liquid handling system. Volumes from 3 to 900 µL using calibrated syringes and fixed tips were evaluated with various solutions, including aluminum hydroxide and phosphate adjuvants, β-casein, sucrose, sodium chloride, and phosphate-buffered saline. The methodology to set up liquid class pipetting parameters for each solution was to split the process in three steps: (1) screening of predefined liquid class, including different pipetting parameters; (2) adjustment of accuracy parameters based on a calibration curve; and (3) confirmation of the adjustment. The run of appropriate pipetting scripts, data acquisition, and reports until the creation of a new liquid class in EVOware was fully automated. The calibration and confirmation of the robotic system was simple, efficient, and precise and could accelerate data acquisition for a wide range of biopharmaceutical applications.

## Introduction

Automated liquid handling systems are commonly used for different laboratory applications in the development of biopharmaceutical products.^1–4^ Sample preparation, serial dilutions, and reagent transfers are all examples of pipetting steps that must be as accurate and precise as possible.^5,6^ The quality of an automated liquid handling workstation is characterized by its high precision and accuracy. Precision is defined as the exactness of volume displacement by one or more pipetting channels. Accuracy is defined as the difference between the actual transferred volume and the target volume. To ensure optimal pipetting performance, every liquid to be pipetted by an automated liquid handling workstation should be carefully calibrated and verified periodically.

TECAN EVOware software has a Liquid Class editor module allowing the customization of pipetting parameters for each pipetted liquid. Around 25 parameters are available to adjust precision. Each of them can be modified to reach the best possible precision for a given solution, but this is not an easy task.^7^ In addition, two important parameters allow for adjusting accuracy: factor and offset. These parameters are constants of the equation *Y* = *aX* + *b*, where *a* is the factor (slope) and *b* the offset (intercept). Moreover, for a specific solution, multiple subclasses with specific pipetting conditions can be defined depending on the volume range (referred to as subclasses),^7^ as precision and accuracy are not necessarily the same at 5 and 500 µL. Adjusting all these parameters manually is tedious, time-consuming, and has a certain economic impact due to the allocation of specialized human resources.^8^ The need to automate this task becomes critical when formulation process development has to be performed in high throughput with a great diversity of solutions.

Typically, the confirmation of dispensed volumes in automated liquid handlers can be carried out by gravimetric, fluorometric, or photometric approaches.^5–14^ The method has to provide a means to quantify both the accuracy and precision for different test liquids.^8^

In this study, we have developed an automated volume calibration process for fixed tips in a TECAN Freedom EVO liquid handling workstation using an integrated balance and densitometer. Optimization was made by screening predefined liquid class with different parameters to adjust precision, and then modifying the offset and factor to adjust accuracy. The process is easy to use, automatically starting TECAN EVOware software and running appropriate pipetting scripts in function of predefined liquid classes. This procedure has been successfully implemented in an automated formulation platform of high-throughput screening, reducing operator workload and saving time. To demonstrate the reliability of this automated process, various solutions or suspensions were selected: aluminum hydroxide (AH) and phosphate (AP) adjuvants, β-casein, sucrose, sodium chloride, and phosphate-buffered saline.

## Materials and Methods

### Materials

β-Casein powder was obtained from Sigma-Aldrich (St. Louis, MO) (density [ρ] = 1.008, T = 22.4 °C). A stock solution of 1 mg/mL β-casein was used. Autoclaved AH (Alhydrogel) and AP (Adjuphos) were obtained from Brenntag (Mülheim/Ruhr, Denmark) at 10.380 and 4.600 mg/mL, respectively (ρ = 1.025, T = 22.7 °C for AP; ρ = 1.018, T = 21.4 °C for AH). Aluminum concentration was expressed in micrograms of aluminum per milliliter, corresponding to 2.89 µg AH/mL and 4.52 µg AP/mL. Sucrose 50% (m/v) was obtained from VWR (Leuven, Belgium) (ρ = 1.190, T = 22.5 °C) and potassium hydrogenophosphate from Calbiochem, containing 150 mM NaCl and 10 mM PO_4_, pH 7.0 (ρ = 1.020, T = 22.1 °C). NaCl, prepared at 1 M, was provided by Merck (ρ = 1.189, T = 26.6 °C). Polypropylene troughs of 100 mL were obtained from TECAN (Männedorf, Switzerland).

### Liquid Handling System

The liquid handling platform was a TECAN Freedom EVO 200 with a Liquid Handling arm (LiHa) with system liquid and mounted with 1 mL syringes and eight standard tips (stainless steel fixed tip with soft Teflon outside coating). The workstation was placed under laminar flow to ensure sterility conditions. Prior to sample delivery, a washing step was performed by the TECAN, unless otherwise noted. Version 2.4 of TECAN EVOware was used. This version offers the advantage that the liquid class file was an XML file format easily editable through a Visual Basic (VB) development.

### Gravimetric Approach

The solution was transferred sequentially from each tip individually (one tip at a time) onto an analytical Sartorius CPA 224S balance (Sartorius AG, Göttingen, Germany) with a 0.1 mg precision to measure the weight of the dispensed liquid. A customized chamber was placed on the balance to limit airflow, which could otherwise interfere with weighing of small volumes (less than 100 µL). Between each measurement, the balance was reset automatically. A VB application was developed to display the weight measured by the balance, automatically start the TECAN EVOware software, and run appropriate pipetting scripts. Liquid density was measured using an Anton Paar DMA35 densimeter (St. Albans, UK) and was used to calculate the correctness of dispensed volumes from each tip. The temperature, humidity, and pressure could also be introduced for information and offline monitoring, as changing environmental conditions could influence the volume delivered.^7^

### Predefined Liquid Class Screening

**Figure 1.**
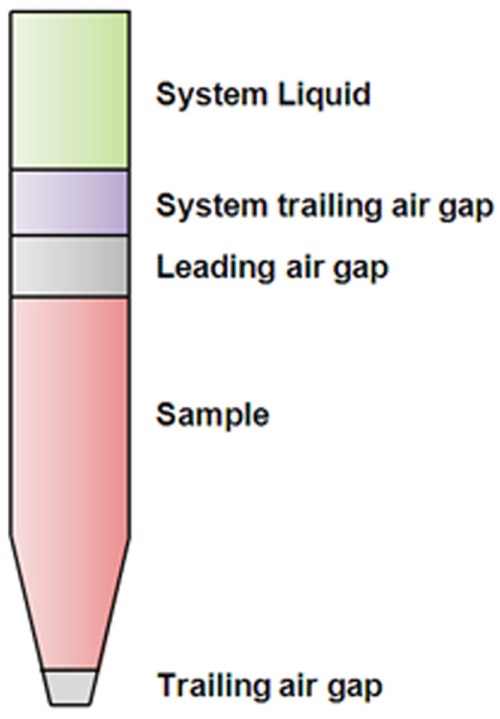
Liquid class air gap setting configurations used for default liquid class.

The TECAN EVOware software includes predefined free dispense liquid classes for different liquid types with different pipetting parameters (**Table 1** and **Fig. 1**): water, serum, DMSO, ethanol, and liquid system. These predefined liquid classes can be split in subclasses that are specific to tip types (fixed and disposable tips) and volume range. For example, predefined liquid class DMSO has three subclasses: the first one from 3.00 to 15.01 µL (subclass 1), the second one from 15.01 to 200.01 µL (subclass 2), and finally, the third one from 200.01 to 1000.01 µL (subclass 3) (**Table 1**). For each subclass, three volumes are tested. For each subclass, different aspiration (aspiration speed, delay, and air gap) and dispensing (dispense speed and break-off speed) parameters are predefined (**Table 1**). The system trailing air gap (STAG) separates the system fluid from aspirated volume. The leading air gap (LAG) is an additional gap between the STAG and the aspirate volume. The trailing air gap (TAG) is the air drawn after the sample at tip opening.^7^

**Table 1.** Configuration Parameters of Predefined Liquid Classes.

	Subclass		Aspiration Parameters					Dispensing Parameters			
Liquid Class	No.	Volumes (µL)	Aspirate speed (µL/s)	Delay (ms)	STAG	LAG	TAG	Dispense Speed	Break-Off Speed (µL/s)	Factor	Offset
DMSO	1	3.00–15.01	20	200	10	10	5	600	150	1.063	0.10
	2	15.01–200.01	100	200	20	0	10			1.100	−0.30
	3	200.01–1000.01	150	300	20	0	10			1.002	19.30
Water	1	3.00–15.01	20	200	10	10	5			1.045	0.20
	2	15.01–500.01	150	200	20	0	10			1.042	−0.04
	3	500.01–1000.01	150	200	20	0	10			1.000	20.26
Serum	1	3.00–15.01	20	200	10	20	10			1.074	0.30
	2	15.01–300.01	100	200	20	0	10			1.060	0.43
	3	300.01–1000.01	100	200	20	0	10			1.007	16.63
Ethanol	1	3.00–1000.01	100	200	20	0	10			1.063	3.4
Liquid system	1	3.00–1000.01	150	200	0	0	10			1.000	0

LAG, leading air gap; STAG, system trailing air gap; TAG, trailing air gap.

Five predefined liquid classes were screened per solution by dispensing at 300 µL. Based on the weight value of the balance, the actual dispensed volume was calculated by the VB application for each of the eight fixed tips using the measured density of the solution introduced by the operator. Based on the precision obtained from each predefined liquid classes, the optimal liquid class type could be selected for subsequent accuracy adjustment by the operator.

**Figure 2.**
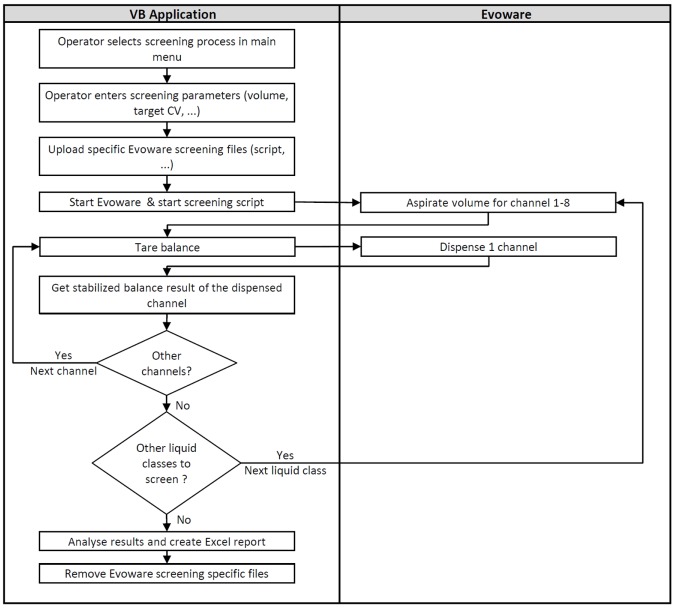
Flow diagram of predefined liquid class screening.

The VB application leads the entire liquid class screening process. First, the operator selects the screening parameters in the user interface and starts the process. Second, the VB application manages the screening procedure by uploading required EVOware files (scripts, labwares, etc.), executing EVOware, and starting the appropriate script automatically. It also includes the balance serial communication handled by the MSComm VB control in order to tare and read data of the balance after dispenses. Data are recorded in an Excel report and shown in the VB application user interface. Third, once the screening process is performed, the VB application analyses results and the liquid classes with the best precision and/or accuracy are highlighted within the Excel report (**Fig. 2**).

### Statistical Parameters and Acceptance Criteria Used in Assessing Performance

Both accuracy and precision are critical for ensuring pipetting performance.^15^ Accuracy was obtained as the percentage of deviation (%*DEV*) and calculated from the actual dispensed volume (*V_A_*) and the intended volume (*V_O_*) (eq 1). Precision was obtained by calculating the coefficient of variation expressed in percentage (%*CV*) and represents the variability between tips and between replicates of a given volume (eq 2). %*CV* was obtained by dividing the standard deviation (*σ*) by the mean (*x*).

$$\mathrm{Accuracy}\left(\%\mathrm{DEV}\right)=\left(\frac{V_{A}-V_{O}}{V_{O}}\right)\times 100$$

$$\mathrm{Precision}\left(\%CV\right)=\frac{\sigma }{x}\times 100$$

In this study, statistical parameters and acceptance criteria were defined in assessing performance for each liquid class and subclass and are presented in **Table 2**.

**Table 2.** Acceptance Criteria Used in Assessing Performance.

	Subclass 1			Subclass 2			Subclass 3		
Volume No.	1	2	3	1	2	3	1	2	3
Serum	15% DEV 3% CV	10% DEV 3% CV	8% DEV 3% CV	7% DEV 3% CV	2% DEV 1% CV	1% DEV 0.75% CV	1% DEV 0.75% CV	0.5% DEV 0.75% CV	0.5% DEV 0.75% CV
DMSO				10% DEV 3% CV	4% DEV 1% CV	2% DEV 0.75% CV			
Water				7% DEV 3% CV	1% DEV 1% CV	0.5% DEV 0.75% CV	0.5% DEV 0.75% CV		

AH, aluminum hydroxide adjuvant; AP, aluminum phosphate adjuvant; CV, coefficient of variation; % DEV, deviation expressed in percent.

### Adjusting Accuracy

After the selection of predefined liquid class in the EVOware software, subclasses were adjusted automatically for three specific volumes (minimum, intermediate, and maximum, referred to as volumes 1, 2, and 3, respectively). Targeted accuracy (**Table 2**) and number of adjustment attempts (in general, maximum 4) were selected in the VB application by the operator. Based on these parameters, the VB application automatically ran the EVOware software and started the appropriate pipetting scripts with the predefined liquid class template, for the volumes to test (**Fig. 2**).

**Figure 3.**
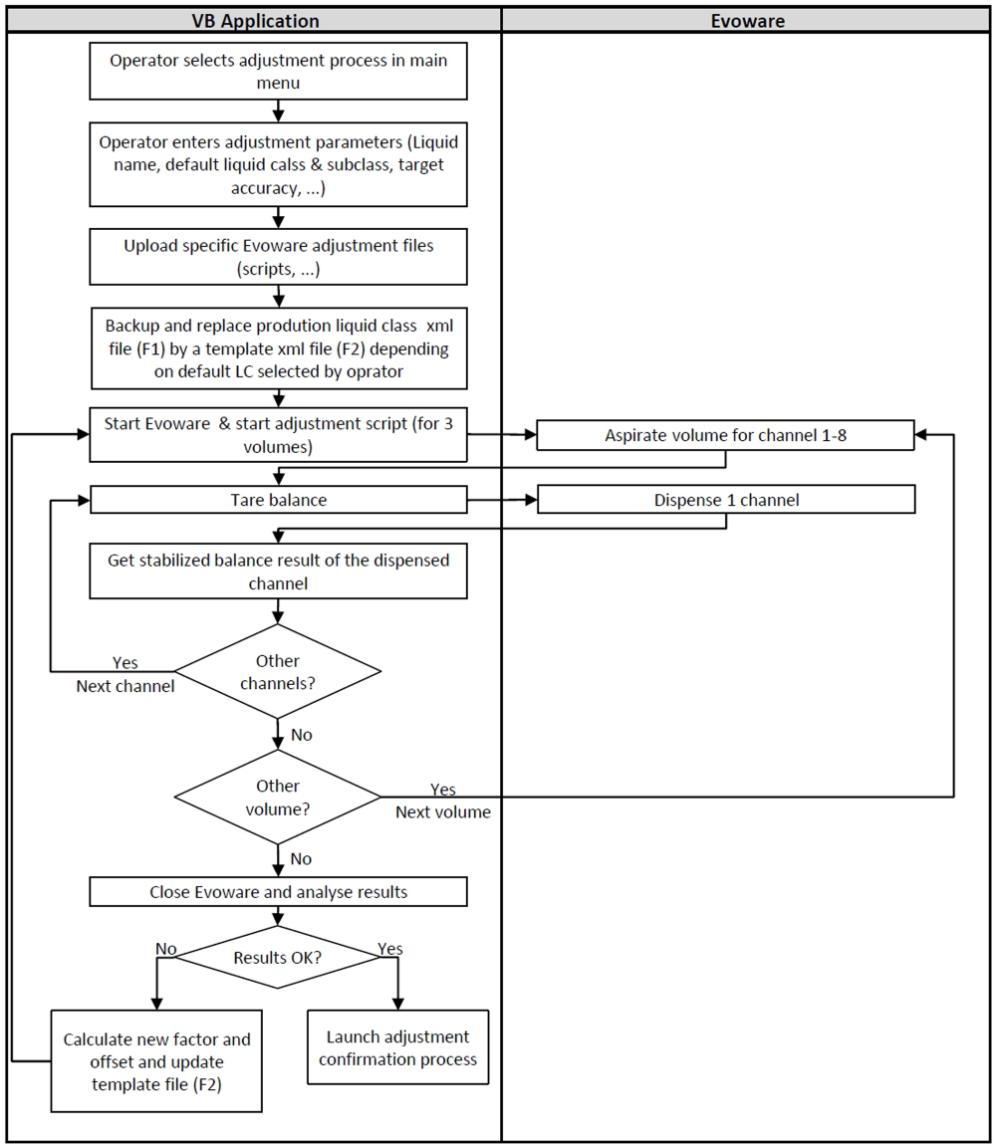
Flow diagram of adjustment.

During the first adjustment, the system adjusted the factor and offset of the subclasses of the predefined liquid class. After measuring all volumes of one subclass (eight data points for each volume with one data point for each channel), the VB application calculated the mean, the precision, and the accuracy of each tested volume. If at least one failed to reach the targeted accuracy, the VB application calculated new factor (eq 3) and offset values (eq 4) (based on the tested factor [*a*_1_] and offset [*b*_1_] values, the slope [*a*_2_] and intercept [*b*_2_] of the linear trend line equation obtained with the theoretical volumes and the calculated average volumes). The VB application exported the new factor and offset values into a temporary TECAN EVOware liquid class file and started a new run for the same three volumes to fine-tune the calibration of the target volume (**Fig. 3**).

$$\mathrm{New Factor}=\frac{a_{1}}{a_{2}}$$

$$\mathrm{New Offset}=\frac{\left(b_{1}-b_{2}\right)}{a_{2}}$$

The VB application performed the adjustments until the three volumes satisfied the targeted accuracy or the number of attempts was reached.

The way to execute the entire adjustment by the VB application is close to the screening process, but for multiple volumes instead of multiple liquid classes, especially for the communication with the balance and the pipetting (**Fig. 3**).

### Confirmation of Factor and Offset

**Figure 4.**
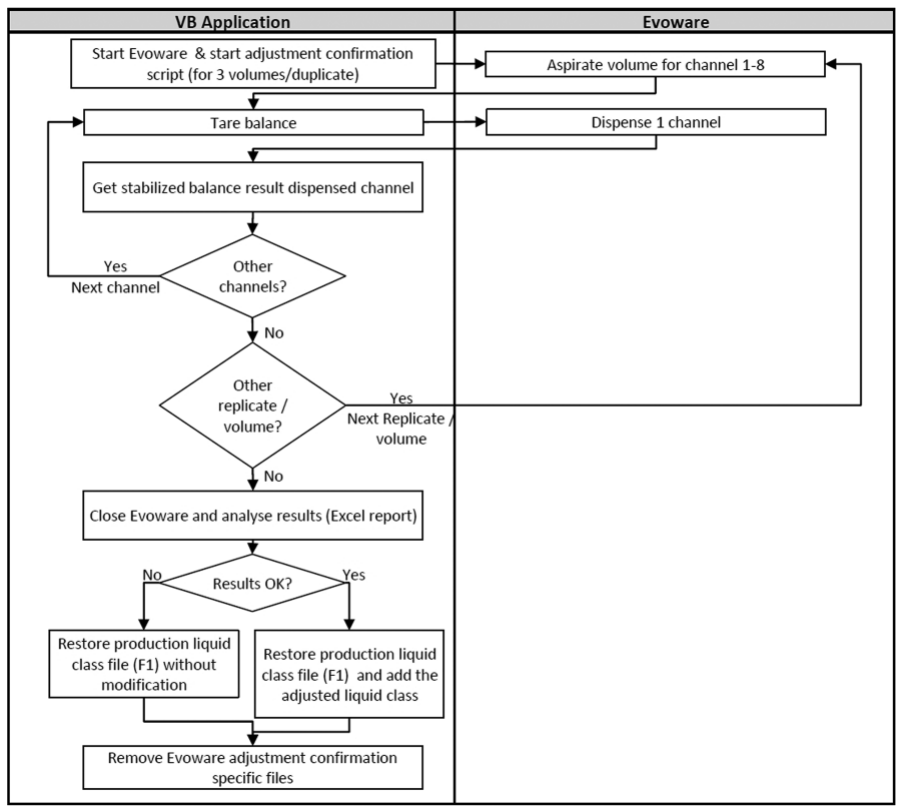
Flow diagram of confirmation process.

If targeted accuracy was reached for the three volumes, the VB application launched a confirmation run of the adjustment (**Fig. 3**). The principle was to confirm that the adjusted factor and offset gave the same results as during the adjustment step, but with a higher number of data points (16 data points for each volume with 2 data point for each channel). Concerning the adjustment step, the VB application calculated the average, precision, and accuracy of each tested volume, which were presented in a report. If targeted accuracy could not be reached for the three volumes, the VB application informed the user. If the accuracy was reached, the parameters were automatically saved by the VB application, adding the new subclass into the EVOware liquid class XML file (**Fig. 4**). Adjustment and confirmation data are recorded in an Excel report available for operators.

The confirmation is the continuity of the adjustment and still entirely managed by the VB application for multiple replicates of the same volumes. The communication with the balance and the pipetting remains identical (**Fig. 4**).

The same procedure (adjustment followed by confirmation) was followed for each subclass of the liquid class.

### Application to the Adsorption Capacity of Aluminum Phosphate Adjuvants

Adsorption capacity of AP for β-casein was determined by performing adsorption isotherms and evaluated using a constant AP concentration (160 µg/mL) and varying β-casein concentrations (475–750 µg/mL).^16^ A sodium chloride solution was used at a final volume of 500 µL in a 96-deep-well plate. The formulations were gently stirred using magnetic bars in a microplate for 18 h at room temperature, using a Vortex Lateral Tumble Stirrer VP 708-CON (V&P Scientific, San Diego, CA). A centrifugation step (2000 rpm for 15 min; Beckman centrifuge GS-6R, rotor type Swing GH 3.8 [Brea, CA]) was performed to obtain aluminum salt-free supernatants. Liquid transfer from the 96-deep-well plate to an ultraviolet-transparent 96-well acrylic microtiter plate (Costar 3679, Corning, New York) was performed using a Liquidator 96. The concentration of β-casein in the supernatant was determined by measuring the optical density at 280 nm in a plate reader (Varioskan Flash Mode Reader, Thermo Fisher Scientific, Grand Island, NY).^17^ Light scattering was corrected by substracting the optical density at 320 nm. Path-length correction was performed by monitoring the intensity of the water absorption peak in the near-infrared region at 975 nm, subtracted for the baseline reading at 900 nm.^17^ The buffer signal was also subtracted. The standard curve for β-casein (0–200 µg/mL) was prepared in 150 mM NaCl.

The Langmuir model was used to estimate the adsorption capacity.^18^ Linear regression was obtained by plotting the protein concentration in the supernatants (expressed in milligrams) divided by the amount of antigen that was adsorbed (total antigen content minus remaining antigen content in the supernatant) per milligram of adjuvant (*y* axis) against the protein concentration in the supernatants (*x* axis). Adsorption isotherms were in agreement with the Langmuir model when linear regression *R*^2^ was higher than 0.99. Adsorption capacity (Cmax) was calculated from the inverse of the slope of the regression line of the Langmuir equation.^19^

### Application to Hydroxyl Content Measurement of Aluminum Hydroxide Adjuvants

6,8-Difluoro-4-methylumbelliferyl phosphate (DiFMUP, D-22065) obtained from Molecular Probes, Inc. (Eugene, OR) was used to determine the relative surface phosphophilicity of AH.^20^ A 10 mM DiFMUP stock solution in DMSO was diluted 20 times with 100 mM 3-(*N*-morpholino)propanesulfonic acid (MOPS) buffer solution (Sigma-Aldrich), pH 7.0 ± 0.1.^20^ The final reaction mixture contained 450 µg/mL AH, 10 mM MOPS buffer, and 50 µM DiFMUP. The Liquidator 96 Manual Benchtop Pipetting System was used for liquid transfer and mixing from the 96-deep-well to the 96-well flat-bottom black opaque Costar plate prior to data acquisition with the fluorescence plate reader. Fluorescence intensity was measured at the upper surface of the samples in a plate reader at 22 °C. Excitation wavelength was set at 358 nm and emission wavelength at 455 nm^20^ (step of 1 nm, excitation bandwidth of 12 nm, and integration time of 100 ms). The kinetics mode setting was 5 min intervals for 60 min to monitor the DiFMUP hydrolysis by AH.^20^ Linear regression was obtained by plotting the fluorescence unit against time (*R*^2^ > 0.99). The catalytic rates were obtained from the slope. The background DiFMUP hydrolysis (DiFMUP without AH) was subtracted. Relative surface phosphophilicity was determined based on the catalytic rate of the aluminum-containing adjuvant raw material (equal to 1).

## Results and Discussion

Automated liquid handling systems are widely used in drug discovery experiments and high-throughput screening processes.^1–4^ The ability of these systems to deliver proper volumes of specific reagents makes them performance tools. Many methods exist to monitor pipetting accuracy and precision, including photometric,^7,8^ fluorimetric,^7,8^ and dual dye photometric approaches.^21–24^ Different authors have reported attempts to automate the process of calibration.^7,9^ The common gravimetric approach has been used with individual vials, removable strips with 8 wells in a 96-well plate holder,^25^ or a 96-well plate format, based on an average across the plate because accuracies of individual wells cannot be determined.^26,27^ In the case of individual containers, they are weighed and manually recorded before and after each filling step. Moreover, water is commonly used as the calibration standard since its density is known at different temperatures. In other cases, the density of solvents was assumed to be equal to that of water (ρ = 1 g/cm^3^) to facilitate the calculations of the volume dispensed by each tip.^5,6^ This process is time-consuming and prone to errors. In contrast, in the colorimetric approach, absorbance is quickly measured in each well of the 96-well microplate in a plate reader. However, this calibration method is complicated because corrections for the correlation between volume and absorbance need to be established for each concentration of the dye used, by performing replicates of manual pipetting and weighing of the 96-well plate before and after addition of the dye. Moreover, automated calibration of fix tips has not been reported yet.

In this study, the gravimetric approach has been used to automate the process of calibration, and was found to be reliable, fast, and easy. The methodology to set up liquid class parameters for each solution was to split the process in three steps. The first step was to identify the parameters for the best precision by screening predefined EVOware software liquid classes. The second step was to adjust the accuracy of the selected liquid class, and the third step was to confirm accuracy and update EVOware software with the calibrated liquid class. Using this methodology, target aliquots of multiple solutions were dispensed, from 3 to 900 µL, with a calibrated syringe and subsequently validated by gravimetric approach. Various sample solutions or suspensions were prepared: AP 4.6 mg/mL, AH 10.38 mg/mL, sucrose 50% (m/v), phosphate-buffered saline (PBS), β-casein 1 mg/mL, and NaCl 1 M.

### Predefined Liquid Class Screening

Performance parameters can vary between different types of liquids due to their physical properties, such as viscosity, surface tension, and density. So, calibration parameters optimized for accurately dispensing water are not optimized for dispensing other types of solution. Different pipetting parameters have been shown to influence performance of dispensing, such as aspirate/dispense rate, pre- and postair gap, and tips.^5,7,8^

Several important factors, such as precision and accuracy, determine the performance of all kinds of liquid handling workstations. Two sets of parameters are related to those parameters for the liquid transfer in the TECAN workstation: pipetting parameters and calibration parameters. Pipetting parameters are more related to precision than accuracy and include factors such as aspiration and dispensing speed, air gap, or contact and noncontact dispensing.^28^ In contrast, calibration parameters are more related to accuracy than precision and define the slope and offset of the calibration curve for a specific liquid class.

Other groups have used the gravimetric approach to calibrate and evaluate the performance of their liquid handling systems based on those parameters of calibration and pipetting.^7,9^ The parameters of calibration were first modified until an acceptable accuracy was achieved, and pipetting parameters were modified in addition to the slope and offset. However, changes in the parameters of calibration can also impact the slope and offset values, and thus can modify accuracy, leading to a new time-consuming step to adjust these calibrating parameters.

**Figure 5.**
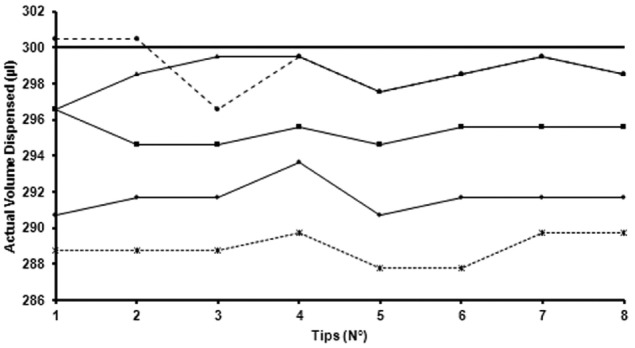
Screening of default liquid class for single dispensing of aluminum phosphate adjuvant using a 300 µL pipetting, with volumes measured gravimetrically. Ethanol (•), DMSO (○), serum (■), water (◊), liquid system (▲).

The novel aspect of the methodology discussed here is, for each liquid to be set up, to screen predefined EVOware liquid classes to identify which one has the optimal parameters for a precise pipetting (**Fig. 5**). For each relevant predefined liquid class, mean, minimum and maximum volumes, %DEV, and %CV are presented for each reagent (**Table 3**, lowest in bold). The liquid class selection is not automatically defined with the best CV because different liquid classes could have a similar precision. However, in case of a very close accuracy, the lowest CV will be preferred. Results from ethanol and liquid system are presented for information due to the lack of different subclasses (**Tables 1** and **3**).

**Table 3.** Screening of Default Liquid Classes.

Solvent		Water-Free Dispense	Serum-Free Dispense	DMSO-Free Dispense	Ethanol-Free Dispense	System Liquid-Free Dispense
AP	Mean volume	291.7	295.4	298.5	298.9	288.9
	Min volume	290.7	294.6	296.6	296.6	287.8
	Max volume	293.7	296.6	299.5	300.5	289.8
	DEV (%)	−2.76	−1.54	**−0.49**	−0.37	−3.70
	CV (%)	0.31	**0.23**	0.35	0.46	0.28
Sucrose	Mean volume	287.4	292.6	294.6	296.6	284.1
	Min volume	285.7	290.8	294.1	295.8	282.4
	Max volume	289.1	294.1	295.8	297.5	285.7
	DEV (%)	−4.20	−2.45	**−1.79**	−1.12	−5.29
	CV (%)	0.35	0.37	**0.21**	0.21	0.37
β-Casein	Mean volume	297.7	301.3	305.4	305.2	287.0
	Min volume	295.7	299.6	303.6	303.4	283.5
	Max volume	299.9	302.6	307.1	306.6	289.9
	DEV (%)	−0.75	**0.45**	1.81	1.74	−4.33
	CV (%)	0.48	**0.41**	0.43	0.38	0.72
PBS	Mean volume	298.5	302.1	305.3	305.8	291.3
	Min volume	297.1	300.0	303.9	302.9	286.3
	Max volume	300.0	303.9	306.9	308.8	298.0
	DEV (%)	**−0.49**	0.69	1.76	1.92	−2.90
	CV (%)	**0.30**	0.40	0.34	0.58	1.56
AH	Mean volume	291.5	296.9	299.2	300.6	289.3
	Min volume	290.8	295.7	297.6	299.6	287.8
	Max volume	291.7	297.6	300.6	301.6	290.8
	DEV (%)	−2.83	−1.03	**−0.25**	0.20	−3.57
	CV (%)	**0.16**	0.29	0.35	0.30	0.36
NaCl	Mean volume	292.1	295.9	299.2	298.9	286.6
	Min volume	290.2	294.4	297.7	297.7	285.1
	Max volume	292.7	296.9	300.3	299.4	287.6
	DEV (%)	−2.65	−1.35	**−0.27**	−0.37	−4.47
	CV (%)	0.30	0.32	**0.29**	0.21	0.38

AH, aluminum hydroxide adjuvant; AP, aluminium phosphate adjuvant; CV, coefficient of variation; % DEV, deviation expressed in % PBS, phosphate buffer saline.

Based on acceptance criteria (**Table 2**), serum was selected for AP and β-casein. DMSO was preferred for sucrose 50% (m/v), AH, and NaCl 1 M, and water for PBS.

### Adjusting Accuracy

Each subclass of liquid class, defined by the screening step, must be adjusted for a specific volume range determined by the VB application. The precisions obtained after the first adjustments are presented in **Tables 4** and **5** for a single delivery of AP. Two adjustments of factor and offset parameters were sufficient to optimize the precision and accuracy in all subclasses for each reagent tested, except in the case of β-casein for subclasses 1, 2, and 3 and PBS for subclass 2, where only one adjustment was sufficient to reach the target accuracy and number of adjustment attempts (**Tables 4** and **5**).

**Table 4.** TECAN Freedom EVO 200 Performance as Measured by the Gravimetric Approach.

Solvent	Default LC	Run	Target Volume	Mean Volume	DEV (%)	CV (%)	Factor (*a*_1_)	Offset (*b*_1_)
AP	Serum	A1	16.0	16.1	0.61	3.24		
		C	16.0	15.9	0.91	2.85		
		A1	158.0	155.9	1.36	0.65	1.060	0.43
		C	158.0	157.9	0.05	0.52	1.075	0.68
		A1	300.0	295.1	1.63	0.25		
		C	300.0	300.1	0.04	0.30		
		A1	301.0	297.6	1.14	0.66		
		C	301.0	299.6	0.45	0.47		
		A1	601.0	600.2	0.13	0.24	1.007	16.63
		C	601.0	601.8	0.14	0.10	1.003	21.36
		A1	900.0	898.3	0.19	0.23		
		C	900.0	899.6	0.04	0.16		
Sucrose	DMSO	A1	16.0	14.5	9.40	4.10		
		C	16.0	17.3	8.32	3.61		
		A1	108.0	101.1	6.43	0.59	1.100	0.30
		C	108.0	104.1	3.61	0.52	1.103	3.67
		A1	200.0	197.2	1.42	0.51		
		C	200.0	201.6	0.79	0.47		
		A1	201.0	198.3	1.33	0.55		
		C	201.0	200.8	0.08	0.45		
		A1	551.0	538.6	2.26	0.15	1.002	19.30
		C	551.0	551.5	0.09	0.21	1.031	16.07
		A1	900.0	877.5	2.50	0.17		
		C	900.0	899.4	0.07	0.20		
β-Casein	Serum	A1	3.0	2.6	13.61	8.54		
		C	3.0	2.8	6.58	8.19		
		A1	9.0	8.5	6.03	1.21	1.074	0.30
		C	9.0	8.7	3.69	2.66	1.078	0.74
		A1	15.0	14.6	2.94	1.49		
		C	15.0	15.1	0.78	1.44		
		A1	16.0	15.5	2.96	8.03		
		C	16.0	15.4	3.82	7.18		
		A1	158.0	159.4	0.87	0.44	1.060	0.43
		C	158.0	158.4	0.28	0.47	1.052	0.75
		A1	300.0	301.6	0.54	0.41		
		C	300.0	299.8	0.07	0.36		
		A1	301.0	302.8	0.60	0.26		
		C	301.0	301.6	0.21	0.26		
		A1	601.0	600.2	0.13	0.21	1.007	16.63
		C	601.0	600.0	0.16	0.24	1.011	14.11
		A1	900.0	899.4	0.07	0.20		
		C	900.0	899.9	0.01	0.19		

AP, aluminum phosphate adjuvant; A, adjustment; C, confirmation; CV, coefficient of variation; % DEV, deviation expressed in % LC, liquid class.

**Table 5.** TECAN Freedom EVO 200 Performance as Measured by the Gravimetric Approach.

Solvent	Default LC	Run	Target Volume	Mean Volume	DEV (%)	CV (%)	Factor (*a*_1_)	Offset (*b*_1_)
PBS	Water	A1	3.0	2.3	22.79	10.84		
		C	3.0	3.2	6.62	10.22		
		A1	9.0	8.0	11.2	8.99	1.045	0.20
		C	9.0	9.2	2.26	4.88	1.027	1.29
		A1	15.0	13.9	7.35	10.76		
		C	15.0	15.0	0.08	1.42		
		A1	16.0	15.8	1.19	3.97		
		C	16.0	16.2	1.10	3.24		
		A1	258.0	256.7	0.49	0.24	1.042	0.04
		C	258.0	257.4	0.25	0.41	1.043	0.40
		A1	500.0	506.9	0.15	0.81		
		C	500.0	498.9	0.22	0.31		
		A1	501.0	499.9	0.22	0.62		
		C	501.0	501.7	0.14	0.89		
		A1	701.0	697.9	0.44	0.24	1.000	20.26
		C	701.0	701.5	0.07	0.26	1.007	18.79
		A1	900.0	895.1	0.54	0.20		
		C	900.0	899.6	0.04	0.25		
AH	DMSO	A1	3.0	2.4	19.78	3.78		
		C	3.0	2.9	3.41	7.69		
		A1	9.0	8.1	10.50	2.52	1.063	0.10
		C	9.0	9.2	1.92	3.62	1.164	0.51
		A1	15.0	13.5	10.20	1.89		
		C	15.0	15.1	0.52	2.26		
		A1	16.0	15.9	0.69	18.61		
		C	16.0	16.9	5.75	1.91		
		A1	108.0	102.7	4.88	1.04	1.100	0.30
		C	108.0	106.2	1.68	2.80	1.094	2.67
		A1	200.0	199.6	0.21	0.93		
		C	200.0	200.5	0.26	0.19		
		A1	201.0	200.3	0.36	0.31		
		C	201.0	199.8	0.61	0.52		
		A1	551.0	550.1	0.59	0.25	1.002	19.30
		C	551.0	550.2	0.14	0.36	1.012	17.31
		A1	900.0	895.87	0.68	0.17		
		C	900.0	899.6	0.05	0.16		
NaCl	DMSO	A1	3.0	2.47	17.65	8.13		
		C	3.0	2.9	2.58	9.20		
		A1	9.0	7.76	13.79	3.79	1.063	0.10
		C	9.0	8.8	2.00	1.39	1.218	0.36
		A1	15.0	12.97	13.51	1.72		
		C	15.0	15.3	1.70	2.34		
		A1	16.0	15.7	2.10	6.38		
		C	16.0	16.0	0.13	3.98		
		A1	108.0	110.7	2.50	0.39	1.100	0.30
		C	108.0	108.1	0.07	0.59	1.072	0.64
		A1	200.0	204.3	2.13	0.56		
		C	200.0	200.0	0.02	0.37		
		A1	201.0	206.5	2.72	0.22		
		C	201.0	202.0	0.48	0.47		
		A1	551.0	544.8	1.13	0.28	1.002	19.30
		C	551.0	548.8	0.40	0.31	1.028	9.00
		A1	900.0	887.8	1.35	0.16		
		C	900.0	900.7	0.07	0.17		

A, adjustment; AH, aluminum hydroxide adjuvant; C, confirmation; CV, coefficient of variation; % DEV, deviation expressed in % LC, liquid class; PBS, phosphate buffer saline.

For subclass 1, the %DEV values for AP, sucrose, and β-casein were in accordance with the criteria of acceptance described in **Table 2**, except for 3 and 9 µL of PBS and all volumes for AH and NaCl 1 M. The %CV values were in agreement with the acceptance criteria, except for all volumes of PBS and 9 µL for NaCl 1 M, even if they remained low. For subclass 2, the %DEV values were lower than the criteria of acceptance except for the third volume for AP (1.63%) and NaCl 1 M (2.13%) and for the second volume of sucrose (6.43%) and AH (4.88%). The %CV fitted the acceptance criteria, except for 16 µL of all liquid classes (between 3.24% and 18.61%) and all volumes tested for AH, even if they remained low. For the three volumes tested in subclass 3, the %DEV values were within 0.07%–2.72% and the %CV values were within 0.15%–0.66%, which was a little bit higher than the acceptance criteria.

With the screening of predefined liquid class, we were able to sample the different tested solutions for three subclasses at different volumes with high accuracy and precision, even if a second adjustment was required to increase accuracy and precision.

### Confirmation of Factor and Offset

Based on the adjustments of subclasses, factor and offset parameters were defined for the confirmation step. Liquid dispensing was highly reproducible and accurate, as indicated by the %CV and %DEV values being less than 10.5% for subclass 1 and less than 1.0% for subclasses 2 and 3 (**Tables 4** and **5**). Precision and accuracy met the specifications, except some %CV of low volumes, even though they were lower than 10.5%.

Before the automated calibration procedure was developed, a manual gravimetric method was used to optimize the liquid classes of the TECAN Freedom EVO. This manual procedure to optimize one liquid class usually represented the weight of about 150 tubes (before and after dispensing), as well as the preparation of tubes, worktables, templates, and reports. This manual procedure usually required at least one full day of workload for the operator. Using the automated optimization procedure, the lead time of the whole process (screening, adjustment, and confirmation steps) was decreased by 30%, with an operator workload decreased by more than 90%.

### Adsorption Capacity of Aluminum Phosphate Adjuvants

Aluminum-containing salts are important adjuvants for many licensed human vaccines.^29–31^ The adsorption of an antigen onto an aluminum-containing vaccine is an important factor for enhancing the immune response.^32,33^ Adsorption isotherms are widely used to characterize the mechanisms by which proteins are adsorbed by aluminum-containing adjuvants.^19,34–36^ β-Casein was selected as the model protein to perform adsorption isotherms with AP.^16^ Preliminary studies showed that adsorption and pH equilibration were achieved after 18 h of incubation with stirring for the mix AP/β-casein (data not shown). The adsorption of negatively charged β-casein (IEP 4.6–5.1) by negatively charged AP (PZC 4.5) at pH 7.0 ± 0.1 fitted the Langmuir model (*R*^2^ > 0.99). The adsorption capacity for β-casein, as derived from the Langmuir equation, was 4.31 mg/mg, in accordance with previous studies.^16^

The reproducibility of the adsorption method was evaluated by measuring the %CV for inter- and intraplates. For that, two 96-deep-well plates, representing 24 distinct isotherms, were used. The Langmuir equation–derived adsorption capacities for the 24 adsorption isotherms are shown in **Table 6**. The intrarun CVs were 2.73% and 4.24%, and the interrun CV was 3.63%. The estimates of adsorption capacities were highly reproducible using the high-throughput screening method, as indicated by CVs less than 5%.

**Table 6.** Adsorption Capacity of Aluminum Phosphate Adjuvants.

	Columns														
	1	2	3	4	5	6	7	8	9	10	11	12	Mean	SD	CV (%)
Plate 1	4.43	4.33	4.32	4.26	4.32	4.34	4.16	4.60	4.29	4.41	4.53	4.29	4.36	0.12	2.73
Plate 2	4.36	4.21	4.61	4.17	4.48	4.39	4.25	4.19	4.15	4.03	4.00	4.36	4.27	0.18	4.24
Mean	4.40	4.27	4.47	4.21	4.40	4.37	4.21	4.39	4.22	4.22	4.26	4.33			
SD	0.05	0.09	0.20	0.07	0.12	0.04	0.06	0.29	0.10	0.27	0.38	0.05			
CV (%)	1.17	2.05	4.58	1.58	2.63	0.83	1.46	6.56	2.38	6.38	8.88	1.17			

CV, coefficient of variation; SD, standard deviation.

### Surface Phosphophilicity of Aluminum Hydroxide Adjuvants

The surface phosphophilicity of AH was determined by monitoring the catalytic rate of DiFMUP mediated by the free hydroxyl groups on the AH surface^20^ by fluorescence spectroscopy. The reproducibility of the surface phosphophilicity method was evaluated by measuring the %CV for inter- and intraplates on 192 wells distributed on two 96-deep-well plates. Results are presented in **Table 7**. The estimates of surface phosphophilicity of AH were highly reproducible, as indicated by intrarun CVs reaching 8.76% and 9.75%, and interrun CV reaching 9.26%.

**Table 7.** Surface Phosphophilicity of Aluminum Hydroxide Adjuvant.

	Columns														
	1	2	3	4	5	6	7	8	9	10	11	12	Mean	SD	CV (%)
Row 1	44.9	45.1	46.9	49.7	44.1	44.9	47.9	42.8	46.3	50.2	54.5	50.5	47.3	3.4	7.1
Row 2	53.1	53.0	53.3	46.6	46.7	54.8	51.6	49.0	47.4	48.6	46.7	45.5	49.7	3.3	6.6
Row 3	48.0	51.6	51.1	48.6	53.4	53.7	57.2	44.8	40.5	51.5	53.4	50.0	50.3	4.4	8.8
Row 4	50.6	51.2	49.3	44.7	50.0	52.0	51.1	54.6	54.3	57.7	54.9	41.5	51.0	4.5	8.8
Row 5	45.1	56.9	54.6	56.6	56.4	54.2	48.2	46.1	50.7	55.1	52.3	52.8	52.4	4.1	7.8
Row 6	53.7	55.0	58.9	43.6	48.7	59.5	54.8	50.6	52.8	55.7	56.4	46.8	53.0	4.8	9.1
Row 7	46.8	53.1	52.1	53.3	55.0	54.0	56.4	43.9	43.1	49.4	53.6	50.6	50.9	4.3	8.5
Row 8	47.5	49.3	48.3	45.4	47.0	50.1	49.6	45.9	48.4	48.1	52.6	49.7	48.5	2.0	4.0
Mean	48.7	51.9	51.8	48.6	50.2	52.9	52.1	47.2	48.0	52.0	53.0	48.4			
SD	3.4	3.6	3.9	4.5	4.4	4.2	3.6	3.9	4.7	3.6	2.9	3.6			
CV (%)	7.0	6.9	7.4	9.2	8.8	7.9	7.0	8.3	9.8	7.0	5.5	7.5			

CV, coefficient of variation; SD, standard deviation.

## Conclusions

Automated gravimetric calibration was used to optimize the accuracy and precision of liquid dispensing with the TECAN Freedom EVO 200 liquid handling system. A novel approach has been designed that uses three steps: screening of predefined liquid class, including different pipetting parameters in the function of various subclasses to increase precision, followed by a series of adjustments of calibration parameters to improve accuracy using density, and finally a confirmation with defined parameters. Using this gravimetric approach, we were able to optimize the dispensing accuracy and precision for six different solvents: AH and AP, β-casein, PBS, sucrose, and sodium chloride. The automated gravimetric approach has the potential to be incorporated into vaccine development, as demonstrated by the various applications, being a robust, rapid, and reproducible method to calibrate liquid handling system. Due to the similarity between TECAN Freedom EVO 200 and other commercially available robotic liquid handlers, this automated process should be adaptable despite engineering and mechanical differences. Moreover, this application could also be adapted to optimize liquid classes with disposable tips by managing through the VB application the tips’ size, depending on the dispensed volume.
